# Rac1-Dependent Lamellipodial Motility in Prostate Cancer PC-3 Cells Revealed by Optogenetic Control of Rac1 Activity

**DOI:** 10.1371/journal.pone.0097749

**Published:** 2014-05-21

**Authors:** Takuma Kato, Katsuhisa Kawai, Youhei Egami, Yoshiyuki Kakehi, Nobukazu Araki

**Affiliations:** 1 Department of Urology, School of Medicine, Kagawa University, Miki, Kagawa, Japan; 2 Department of Histology and Cell Biology, School of Medicine, Kagawa University, Miki, Kagawa, Japan; Southern Illinois University School of Medicine, United States of America

## Abstract

The lamellipodium, an essential structure for cell migration, plays an important role in the invasion and metastasis of cancer cells. Although Rac1 recognized as a key player in the formation of lamellipodia, the molecular mechanisms underlying lamellipodial motility are not fully understood. Optogenetic technology enabled us to spatiotemporally control the activity of photoactivatable Rac1 (PA-Rac1) in living cells. Using this system, we revealed the role of phosphatidylinositol 3-kinase (PI3K) in Rac1-dependent lamellipodial motility in PC-3 prostate cancer cells. Through local blue laser irradiation of PA-Rac1-expressing cells, lamellipodial motility was reversibly induced. First, outward extension of a lamellipodium parallel to the substratum was observed. The extended lamellipodium then showed ruffling activity at the periphery. Notably, PI(3,4,5)P_3_ and WAVE2 were localized in the extending lamellipodium in a PI3K-dependent manner. We confirmed that the inhibition of PI3K activity greatly suppressed lamellipodial extension, while the ruffling activity was less affected. These results suggest that Rac1-induced lamellipodial motility consists of two distinct activities, PI3K-dependent outward extension and PI3K-independent ruffling.

## Introduction

Cell migration plays an important role in embryonic organogenesis; wound healing and immune responses; and the pathogenesis of several diseases including cancer invasion and metastasis [1], [2]. Therefore, an understanding of the molecular mechanisms underlying cell migration is important for developing new therapeutic strategies for preventing tumor invasion and metastasis. Cell migration involves the processes of polarized cellular protrusion and adhesion in the direction of movement, cell contraction, disassembly of adhesive foci, and retraction at the periphery of the cell’s trailing edge [1]. During the tumor cell migration that is associated with cancer metastasis and invasion, metastatic cells exhibit drastic changes in shape. This deformation is caused by actin cytoskeletal remodeling, which is regulated by Rho family GTPases such as Cdc42 and Rac1. Rho family GTPases behave as molecular switches, cycling between active GTP-bound forms and inactive GDP-bound forms. Rho family GTPases are activated by guanine nucleotide exchange factors (GEFs) and inactivated by GTPase-activating proteins (GAPs) [3]. Rac1, a member of the Rho family GTPases, leads to the production of sheet-like protrusions referred to as lamellipodia or membrane ruffles, while Cdc42, another member of the Rho family, creates spike-like protrusions called filopodia [3]. Rac1 is hyperactivated in metastatic prostate cancer cells [4]. Additionally, the inhibition of Rac1 activity blocks the migration and invasion of prostate cancer cells [5]. These studies suggest that Rac1-mediated lamellipodial formation plays an important role in prostate cancer metastasis.

To date, the expression of Rac1 mutants such as the constitutively active (CA) Rac1Q61L and the dominant negative (DN) Rac1T17N has been widely used for investigating the involvement of Rac1 in lamellipodial formation and ruffling [6]. However, the cell phenotype data obtained using Rac1 mutants must be interpreted with caution. Due to the effects of irreversible, permanent and global expression in the cells, it is hard to say that the phenotypes of cells expressing Rac1 mutants exactly reflect the protein’s action as a molecular switch. To elucidate the precise role of the spatiotemporal activation of Rac1, Wu et al. [7], [8] recently developed a photo-activatable Rac1 (PA-Rac1) system by fusing a light-oxygen-voltage (LOV) domain and a carboxy-terminal helical extension (Jα) sequence to the amino terminus of a constitutively active Rac1. LOV is a protein light-switch domain of *Avena sativa* phototropin 1. In the dark, the flavin-binding LOV domain interacts with Jα and blocks the effector binding site of PA-Rac1 by configuring into its closed conformation. Irradiation with light at 400–500 nm light induces the dissociation of LOV domain and Jα helix, and leads to Rac1 activation. This photo-induced activation is reversible. Using this system, localized Rac1 activation was shown to be sufficient to induce cell motility and determine the direction of cell movement [7], [8].

The relationship between Rac1 and phosphatidylinositol 3-kinase (PI3K) in the formation of lamellipodia is complicated because PI3K functions both upstream and downstream of Rac1 [9]. Phosphatidylinositol 3,4,5-triphosphate (PI(3,4,5)P_3_) is known to be bind Rac GEFs and then accelerate actin polymerization through Rac1 activation [10]. Additionally, a positive feedback loop has been reported between PI(3,4,5)P_3_ and Rac for cell polarity during eukaryotic chemotaxis [11], [12]. However, in the regulation of cell protrusion and polarity, reports regarding the function of PI3K downstream of Rac1 are mixed [13], [14]. Thus, the precise role of PI3K downstream of Rac1 remains a controversial issue.

To clarify the relevance of PI3K to Rac1-dependent lamellipodial motility, we applied the PA-Rac1 system to prostate cancer cells. Photomanipulation of PA-Rac1 activity using a blue laser enabled us to distinguish two lamellipodial motile processes in living cells: lamellipodial extension and peripheral ruffling. Notably, we found that PI3K inhibitors suppressed the initiation of lamellipodial extension but had little effect on peripheral ruffling. The present study revealed that Rac1-dependent lamellipodial motile processes consist of two dissociable activities: PI3K-dependent lamellipodial outward extension and PI3K-independent peripheral ruffling.

## Materials and Methods

### Reagents and cDNA Constructs

Fetal bovine serum (FBS) and RPMI-1640 were obtained from the Sigma Chemical Co. (St. Louis, MO). The X-tremeGENE HP DNA Transfection Reagent was acquired from Roche Diagnostic Systems (Basel, Switzerland). The other reagents were purchased from Wako Pure Chemicals (Osaka, Japan) or Nacalai Tesque (Kyoto, Japan), unless otherwise indicated.

pTriEx/mCherry-PA-Rac1Q61L (plasmid #22027) and pTriEx/mCherry-PA-Rac1 T17N (plasmid #22029) were obtained from Addgene (Cambridge, MA). Dr. Joel A. Swanson (University of Michigan) kindly provided the pmCitrine-AKT-pleckstrin homology domain (PH) and pmCitrine-Rac1Q61L. The pEGFP-N1-WAVE2 constructs were generous gifts of Dr. Tadaomi Takenawa (Kobe University).

### Cell Culture and Transfection

PC-3 human prostate cancer cells were purchased from the American Type Culture Collection (Rockville, MD) and were maintained in RPMI medium containing 10% heat-inactivated FBS, 100 U/ml penicillin, and 100 µg/ml streptomycin. The cells were maintained at 37°C in a humidified 5% CO_2_ incubator. For live-cell imaging, the cells were seeded on 25 mm coverslips in 35 mm dishes at a density of 2.0×10^4^ cells/dish and were incubated overnight before transfection.

The X-tremeGENE HP DNA Transfection Reagent was used for plasmid transfection according to the manufacturer’s instructions. pTriEx/mCherry-PA-Rac1Q61L was added to the 35 mm dishes. In the co-transfection treatments, 0.01–0.5 µg of the appropriate plasmids was added to the 35 mm dishes together with 0.3 µg of the PA-Rac1 plasmid.

### Drug Treatments

To determine the role of PI3K in Rac1-induced cell motility, the cells were treated with PI3K inhibitors during irradiation. We used 50 µM LY294002 (Sigma), 100 nM wortmannin (Sigma), and 1 µM ZSTK474 (Active Biochemicals) as PI3K inhibitors. LY294002 and wortmannin, which are pan-PI3K inhibitors, are widely used as tools for investigating diverse signal transduction processes involving PI3K. ZSTK474 also inhibits all four PI3K isoforms but does not inhibit PI3K-related kinases such as mTOR and DNA-dependent protein kinase. These inhibitors were dissolved in dimethyl sulfoxide (DMSO), stored at −20°C, and applied to the cells at the indicated final concentrations. These inhibitors were usually added to cells 30 min before photoactivation, but in some experiments, they were added during photoactivation. For the control treatments, 0.1% DMSO was applied to the cells.

### Photoactivation and Live-cell Imaging

At 12–24 hours after transfection, the culture medium was replaced with Ringer’s buffer (RB) consisting of 155 mM NaCl, 5 mM KCl, 2 mM CaCl_2_, 1 mM MgCl_2_, 2 mM Na_2_HPO_4_, 10 mM glucose, 10 mM HEPES pH 7.2, and 0.5 mg/ml bovine serum albumin. The 25 mm cover slips were placed in an RB-filled chamber on a 37°C thermo-controlled stage (Tokai Hit INU-ONI, Shizuoka, Japan). Photoactivation of PA-Rac1 and live-cell imaging were performed using an Axio Observer Z1 inverted microscope equipped with a laser scanning unit (LSM700, Zeiss), as previously described [15]. To photoactivate PA-Rac1, the indicated area of the prostate cancer cells expressing mCherry-PA-Rac1 was repeatedly irradiated using a 5 mW 445 nm laser at 0.2% power for the indicated periods in a photobleaching mode. Live-cell images were acquired through a 63x Plan-Apochromat/N. A. 1.4 lens every 15 sec using a 10 mW 555 nm laser at 0.5%–2.0% power to obtain mCherry fluorescence and bright-field phase-contrast images. To visualize PI(3,4,5)P_3_ or WAVE2, the cells were cotransfected with pmCherry-PA-Rac1 and the pmCitrine-AKT-PH domain or pEGFP-WAVE2, respectively. EGFP or mCitrine fluorescence images were acquired only at the first and last time points to avoid unintended photoactivation by the 488 nm laser, as this excitation wavelength slightly overlaps with the photoactivation spectrum [8]. We adjusted the power of 488 nm laser as low as possible, so that the acquisition of the first frame by the laser would not impact PA-Rac1 activity.

Time-lapse images using phase-contrast and fluorescence microscopy were taken at 15 sec intervals and assembled into QuickTime movies using Zen 2009 software (Carl Zeiss). Kymograph analyses were performed using MetaMorph imaging software (Molecular Devices). The image data presented here are representative of the results of at least three independent experiments.

### Quantitative Image Analysis

For quantitative image analysis of the lamellipodial extension due to PA-Rac1 photoactivation in the absence or presence of PI3K inhibitors, we took measurements of cell area increases by subtracting the areas of the cells before photoactivation from those after photoactivation using MetaMorph imaging software.

For quantitative analysis of mCitrine-AKT-PH and EGFP-WAVW2 recruitment to photoactivated areas, the fluorescence intensities of the regions of interest were measured using MetaMorph imaging software. The fluorescence intensities of mCitrine and EGFP after 5 min of photoactivation were compared with the fluorescence intensities in the corresponding area before photoactivation using at least 16 cells.

To quantify the effects of PI3K inhibitors on extended lamellipodia and ruffling activity in mCitrine-Rac1Q61L-expressing cells, the cells were fixed with 4% paraformaldehyde at 30 min after the addition of the PI3K inhibitors (50 µM LY294002, 100 nM wortmannin, or 1 µM ZSTK474) or the vehicle only (0.1% DMSO). Using the MetaMorph imaging system, the maximum diameters of the cells expressing Rac1Q61L were measured before and after the drug treatments to evaluate the effect of PI3K inhibition on the extended lamellipodial. Similarly, the peripheral ruffles per cell were counted using a fluorescence microscope to evaluate the impact of PI3K inhibition on ruffling activity. Thirty cells in each group were subjected to the quantitative image analysis.

Data are presented as the means ± standard error (SE) for the number of cells indicated in the text. Statistical analysis was performed using the Wilcoxon t-test feature of Excel 2012. Differences between the analyzed samples were considered significant at p<0.05.

## Results

### Local Activation of PA-Rac1 Reversibly Induces Lamellipodial Extension and Ruffling

**Figure 1:**
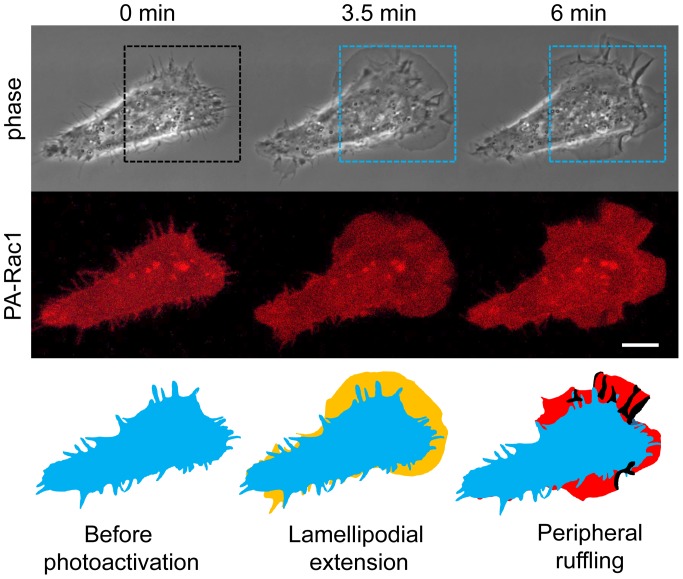
Photo-manipulation of PA-Rac1 induces lamellipodial extension and subsequent ruffling. PC-3 cells were transiently transfected with pTriEx/mCherry-PA-Rac1 and subjected to local photoactivation of PA-Rac1 (rectangular area outlined by blue dots). Time-lapse images of a PC-3 cell expressing mCherry-PA-Rac1 were acquired during photoactivation using 445-nm laser irradiation. The upper and middle panels show phase-contrast and mCherry fluorescence images, respectively. Elapsed times after the initiation of photoactivation are shown at the top. In the bottom panel, the contours of the cell shape at the indicated elapsed times are drawn in blue (0 min, original), yellow (3.5 min, extension phase), and red (6 min, ruffling phase). The black profiles indicate the membrane ruffles. Scale bar, 10 µm.

To elucidate the relationship between Rac1 activation and lamellipodial dynamics, we introduced mCherry-fused PA-Rac1 into PC-3 cells. After confirming the expression of mCherry-PA-Rac1 based on the mCherry signal, we irradiated a peripheral region of the cells using a 445 nm laser during the intervals of image acquisition and acquired phase-contrast images of the live cells every 15 sec by confocal microscopy. Typically, after 1–4 min of irradiation with the 445 nm laser, a thin sheet-like protrusion (i.e., a lamellipodium) extending parallel to the substratum was observed at the cell peripheral site that was irradiated by the 445 nm laser. The lamellipodium reached its maximum length after 5–6 min of PA-Rac1 activation. At that time, following the outward extension, the fully extended lamellipodium curled up its leading edge to show a peripheral ruffling movement. The ruffling movements continued for the duration of irradiation. After irradiation ceased, both the lamellipodial extension and the peripheral ruffling promptly receded (Fig. 1 and Movie S1). In our previous study, dorsal ruffling was induced in RAW 264 macrophages by PA-Rac1 activation [15], [16], but dorsal ruffling was not prominent in PC-3 cells.

**Figure 2:**
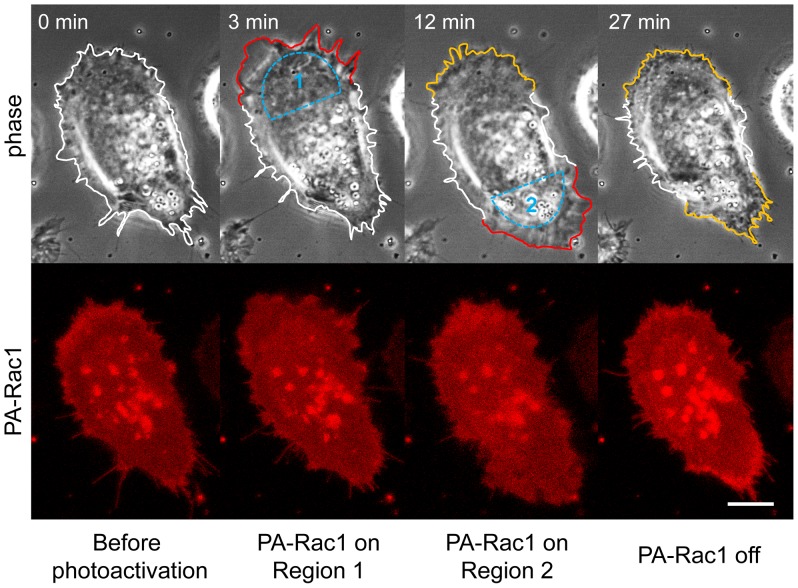
Local and reversible control of lamellipodial dynamics by photomanipulation of PA-Rac1 activity. Time-lapse images of a PC-3 cell expressing mCherry-PA-Rac1 were acquired during PA-Rac1 photo-manipulation by local laser irradiation of different areas. Selected phase-contrast and mCherry fluorescence images are shown. First, region 1 was irradiated for 10 min. The irradiation was then moved to region 2. At 25 min, the irradiation was turned off. Selected time-lapse images of phase-contrast and mCherry fluorescence are shown. The extending and retracting lamellipodia are outlined in red and yellow, respectively. Scale bar, 10 µm.

When we irradiated a different area of the same cell, a lamellipodial extension was generated at the newly irradiated area, suggesting that these phenomena were dependent on local PA-Rac1 activation by light irradiation (Fig. 2). The overexpression of a GDP-bound (dominant negative) mutant of PA-Rac1 (PA-Rac1 T17N) did not induce either lamellipodial extension or peripheral ruffling after 445-nm laser irradiation (not shown). This finding indicated that the morphological changes induced by 445-nm laser irradiation are dependent on GTP-loaded Rac1.

### PA-Rac1-induced Lamellipodial Extension is Dependent on PI3K

**Figure 3:**
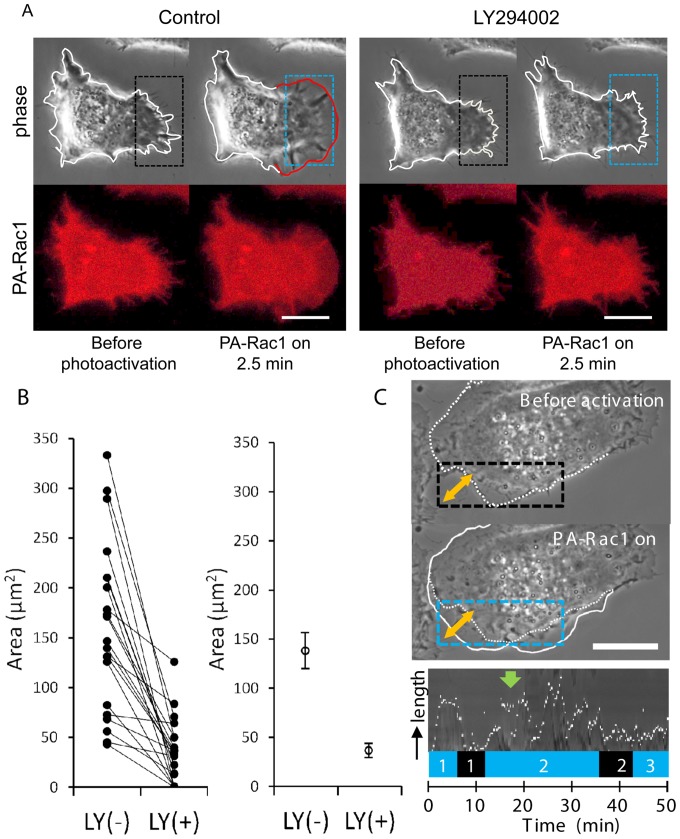
PI3K is required for lamellipodial extension but not for peripheral ruffling. (A) PC-3 cells were transiently transfected with pTriEx/mCherry-PA-Rac1. The cells were subjected to repeated photoactivation in the absence (control) or presence of 50 µM LY294002. The leading edge of the extending lamellipodium is outlined in red. Scale bar, 10 µm. (B) The increased cell area due to lamellipodial extension was quantified by subtracting the area of the cell before photoactivation from the area of the cell 7 min after the beginning of PA-Rac1 activation. This increase was verified in 22 PC-3 cells. The data plot shows the increased area due to lamellipodial expansion that was induced by PA-Rac1 photoactivation in the absence [LY(−)] or presence of LY294002 [LY(+)]. The significance of the differences between LY(−) and LY(+) was confirmed with the Wilcoxon t-test (right). The increase in the lamellipodial area in the presence of LY294002 was significantly lower than that of the control cells (p<0.01). (C) Kymographic analysis was performed at a two-headed arrow placed across the lamellipodium of a PC-3 cell expressing mCherry-PA-Rac1 before and after the addition of LY294002. The laser-irradiated area is indicated with a blue rectangle. The white line outlines the extending lamellipodium, and the dotted line outlines the original cell shape. The lower panel shows the kymograph of a lamellipodium undergoing changes in length. The kymograph demonstrates the extension and retraction of a lamellipodium during PA-Rac1 activation (blue 1) and deactivation (black 1), respectively. The green arrow indicates the addition of LY294002. The PI3K inhibitor had less of an inhibitory effect on lamellipodial extension and ruffling (blue 2). However, the initiation of lamellipodial extension was drastically inhibited (black 2–blue 3). Scale bars, 10 µm.

To examine the effect of inhibiting PI3K activity on PA-Rac1-induced lamellipodial motility, we used LY294002, a synthetic inhibitor of PI3K. We first confirmed that the cells expressing PA-Rac1 exhibited lamellipodial extension and ruffling due to photoactivation. After ceasing photoactivation, we added 50 µM LY294002 to the same cells. When we irradiated the same regions of the cells 30 min after the addition of LY294002, lamellipodial extension was greatly suppressed by the PI3K inhibitor (Fig. 3A and Movie S2). We performed the same experiments using 22 PC-3 cells and quantitatively compared the area increase due to PA-Rac1 activation in each cell before and after treatment with LY294002 (Fig. 3B). The quantitative image analysis demonstrated that the increase of cell area due to PA-Rac1-induced lamellipodial extension was significantly suppressed by LY294002 (p<0.01, n = 22, Fig. 3B).

Additionally, we conducted kymographic analysis to determine the changes in length of the lamellipodia. After confirming PA-Rac1-induced lamellipodial extension and ruffling due to PA-Rac1 photoactivation, we added LY294002 to the cells during PA-Rac1 photoactivation. Although the lengths of the lamellipodia were changed due to peripheral ruffling, the inhibitory effect of LY294002 on lamellipodial extension was small. Peripheral ruffling persisted for the duration of irradiation. Immediately after photoactivation ceased, both lamellipodial extension and peripheral ruffling receded completely. When we re-irradiated the same region, the cells did not show lamellipodial extension (Fig. 3C). These results suggest that the formation of a lamellipodium is more sensitive to PI3K inhibitors than is the maintenance of extended lamellipodia and peripheral ruffling.

Similar results were obtained using 100 nM wortmannin or 1 µM ZSTK474 as PI3K inhibitors (Fig. S1). As a control, the same PA-Rac1 activation experiments were performed using cells treated with 0.1% DMSO, the vehicle of the inhibitors. PA-Rac1-induced lamellipodial formation was not inhibited by DMSO (Fig. S2).

### PA-Rac1 Photoactivation can Locally Activate PI3K and Recruit WAVE2

**Figure 4:**
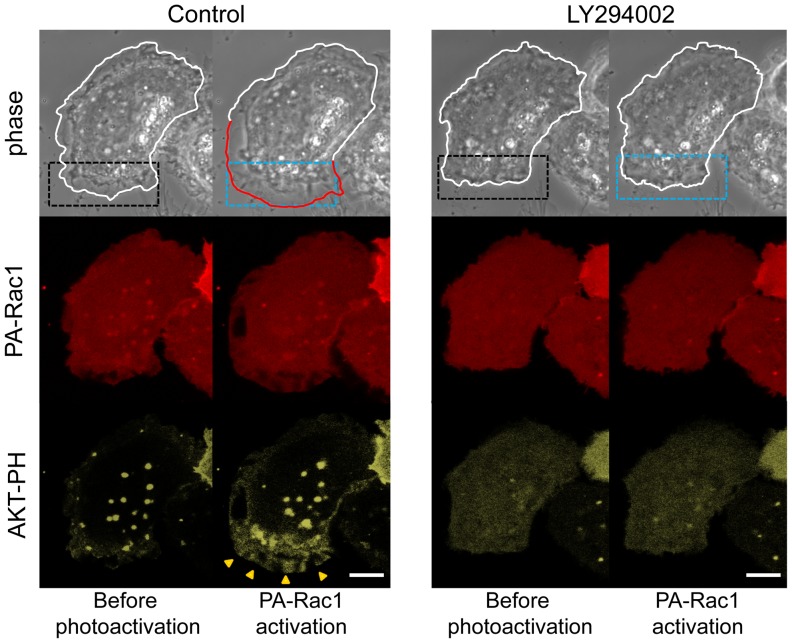
PI(3,4,5)P _3_ in lamellipodia after PA-Rac1 photoactivation in the absence or presence of LY294002. PC-3 cells were co-transfected with pTriEx/mCherry-PA-Rac1 and mCitrine-AKT-PH. Phase-contrast, mCherry-PA-Rac1 (red fluorescence), and mCitrine-AKT-PH (yellow fluorescence) images were acquired before and after PA-Rac1 photoactivation. PA-Rac1 photoactivation was repeated in the same cell region in the absence (control) or presence of 50 µM LY294002. The levels of PI(3,4,5)P_ 3_ were increased in the extending lamellipodium by photoactivation (arrowheads). In the presence of LY294002, PI(3,4,5)P_3_ was not increased in the region where PA-Rac1 was photo-activated. The blue-dotted rectangle indicates the photoactivation area. The extending lamellipodium is outlined in red. Scale bars, 10 µm.

Because PA-Rac1-induced lamellipodial extension was inhibited by the PI3K inhibitors, we attempted to clarify that PA-Rac1 activation led to PI3K activation. To monitor the production of PI(3,4,5)P_3_ by PI3K activity, PC-3 cells were cotransfected with pmCitrine-AKT-PH and pTriEx/mCherry-PA-Rac1Q61L and observed using a Zeiss LSM700 (Fig. 4). The fluorescence intensity of mCitrine-AKT-PH at the lamellipodial leading edge after 5 min of PA-Rac1photoactivation was measured using MetaMorph imaging software and was quantitatively compared with that of the same region before photoactivation. After 5 min of local activation of PA-Rac1, the fluorescence intensity of mCitrine-AKT-PH showed a 125.4% ±22.3% increase (n = 16) compared with that measured before photoactivation, suggesting that the levels of PI(3,4,5)P_3_ in the extending lamellipodia were greatly increased by the local photoactivation of PA-Rac1. In the presence of LY294002, no cells showed an increase in the fluorescence intensity of mCitrine-AKT-PH after irradiation. This finding suggests that Rac1 photoactivation activates PI3K to produce PI(3,4,5)P_3_ from PI(4,5)P_2_ at the membrane of the extending lamellipodia.

**Figure 5:**
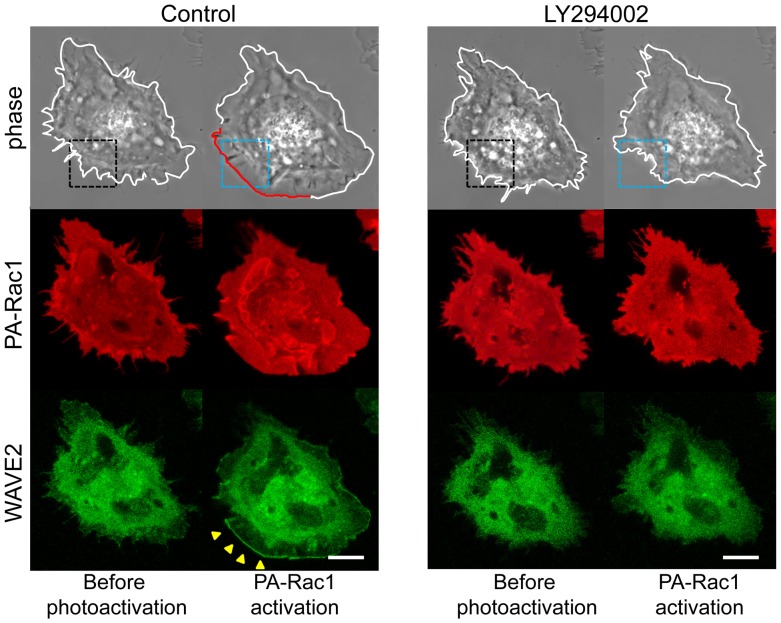
PI3K-dependent WAVE2 recruitments to the leading edge of the extending lamellipodium during PA-Rac1 activation. PC-3 cells were co-transfected with pTriEx/mCherry-PA-Rac1 and pEGFP-N1-WAVE2. Phase-contrast, mCherry-PA-Rac1 (red fluorescence), and EGFP-WAVE2 (green fluorescence) images were acquired before and after PA-Rac1 photoactivation. PA-Rac1 photoactivation was repeated in the same cell region in the absence (control) or presence of 50 µM LY294002. The yellow arrowheads indicate that WAVE2 was recruited to the leading edge of the extending lamellipodium. In the presence of LY294002, WAVE2 was not recruited to the periphery of the cells where PA-Rac1 was photoactivated. The blue-dotted rectangle indicates the photoactivation area. Scale bar, 10 µm.

Furthermore, we examined the dynamics of WAVE2 during the lamellipodia-generating process, because WAVE2 plays a major role in Rac1-induced actin reorganization in association with PI(3,4,5)P_3_ [17]–[19]. After 5 min of irradiation with 445-nm light, EGFP-WAVE2 localized as a dotted line at the leading edge of the extending lamellipodial (Fig. 5). The fluorescence intensity of EGFP-WAVE2 after photoactivation showed a 315.1% ±54.4% increase (n = 17) at the leading edge of the extending lamellipodia. After the addition of LY294002, neither EGFP-WAVE2 recruitment nor lamellipodial extension was induced by PA-Rac1photoactivation (Fig. 5). These findings indicate that WAVE2 is recruited by PI(3,4,5)P_3_ and contributes to Rac1-dependent lamellipodial extension through actin polymerization.

### PI3K Inhibitors do not Affect Extended Lamellipodia but do Enhance Peripheral Ruffling

**Figure 6:**
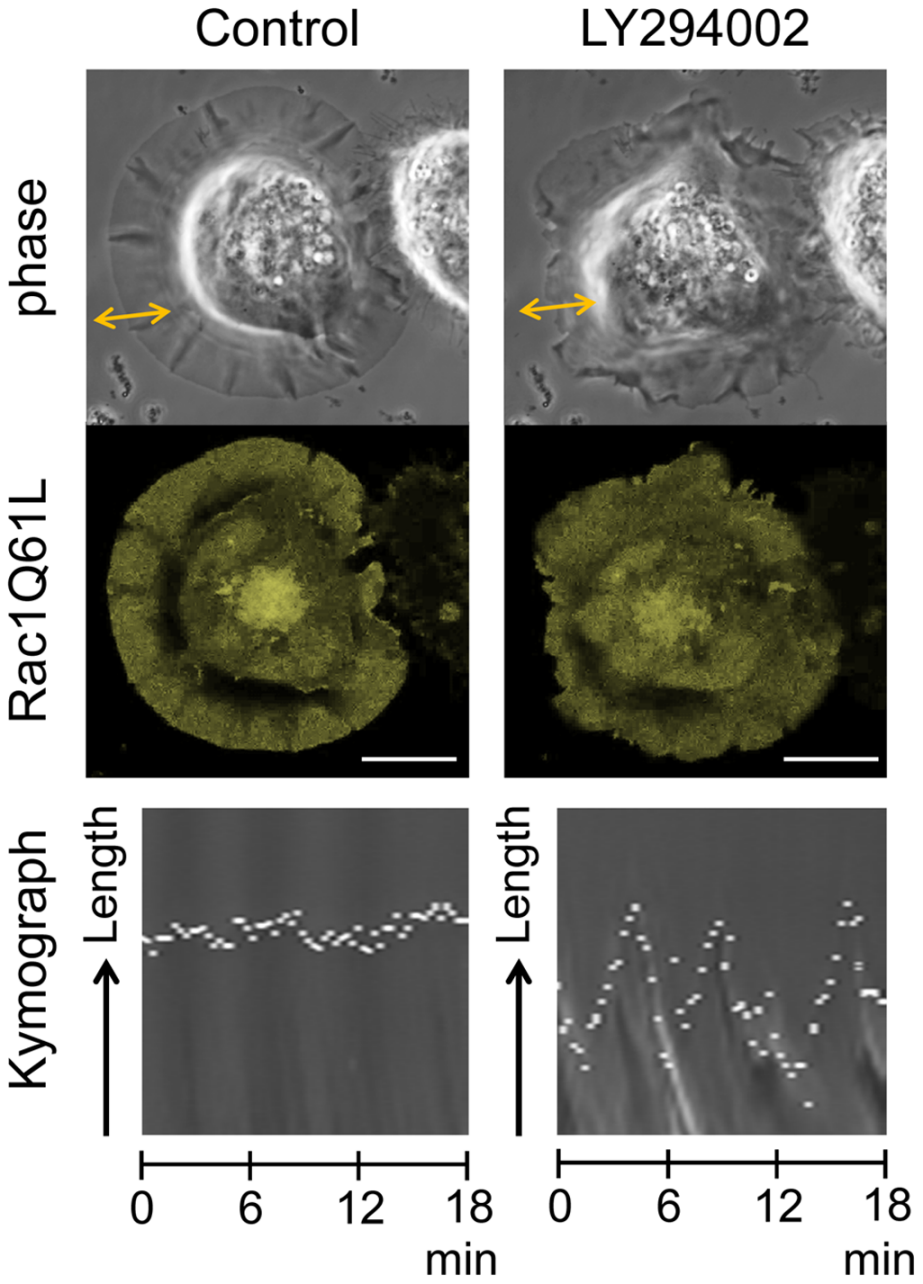
Effect of LY294002 on the extended lamellipodial motility in PC-3 cells expressing constitutively active Rac1Q61L. PC-3 cells were transfected with pmCitrine-Rac1Q61L. The time-lapse images of phase-contrast and mCitrine-Rac1Q61L fluorescence were captured before (left) and after (right) the addition of LY294002. Kymographs were created to show the changes in length of a lamellipodium at the position of the two-headed line. The mCitrine-Rac1Q61L-expressing cell had an extended lamellipodium around its entire circumference. After the addition of 50 µM LY294002, the extended lamellipodium had shrunk only slightly, but the peripheral ruffling was pronounced. The kymographs show dynamic changes in length due to enhanced ruffling after the addition of LY294002. Scale bars, 10 µm.

To clarify the effect of PI3K inhibition on the maintenance of extended lamellipodia, we applied LY294002 to PC-3 cells expressing mCitrine-Rac1Q61L, a constitutively active Rac1 mutant. The mCitrine-Rac1Q61L-expressing cells had well-spread lamellipodia around their entire circumferences. When we added 50 µM LY294002 to these cells, the extended lamellipodia shrank only slightly, even after 30 min. Surprisingly, peripheral ruffling activity was markedly enhanced by the PI3K inhibition in the Rac1Q61L-expressing cells (Fig. 6 and Movie S3). Quantitative analysis of the changes in maximum cell diameter indicated that this factor was not significantly affected by any PI3K inhibitor, whereas the number of peripheral ruffles was greatly increased after 30 min of PI3K inhibition (Table 1).

**Table 1:** Effects of PI3K inhibitors on extended lamellipodia and peripheral ruffles in Rac1Q61L expressing PC-3 cells.

	Cell diameter ± SE (µm) a	Number of ruffles ± SE b
Control	50.8±2.8	1.2±0.3
0.1% DMSO	49.0±1.5	1.3±0.3
50 µM LY294002	56.4±2.3	2.9±0.4*
100 nM wortmannin	49.5±2.3	3.0±0.4*
1 µM ZSTK474	47.9±1.5	2.4±0.3*

Thirty mCitrine-Rac1Q61L expressing cells in each group were subjected to quantitative image analysis. After 30 min of the indicated drug treatments, the cells were fixed with 4% paraformaldehyde, rinsed in PBS and observed by fluorescence microscopy.

a The maximum diameter of each cell was measured using MetaMorph.

b The number of peripheral ruffles per each cell was counted. Data are the means ± SE (n = 30).

*p<0.01 compared to the control.

## Discussion

Lamellipodia can be classified into three types: the thin leading edge of a cell that extends the membrane along the substratum, the peripheral ruffles formed by the upward bending of the leading edge, and the vertical dorsal ruffles that appear behind the leading edge on the dorsal surface of the cell [9], [20]. However, the different mechanisms that regulate these lamellipodial motile processes have not been clarified. PC-3 cells and other prostate cancer cells do not exhibit dorsal ruffling, which is observed in RAW264 macrophages after PA-Rac1 activation [15]. This discrepancy is most likely due to the differences between these cell types. PC-3 cells showed remarkable lamellipodial extension and peripheral ruffling upon PA-Rac1 activation. The present study was undertaken to characterize lamellipodial dynamics and their regulation in PC-3 cancer cells, as lamellipodial motility plays a central role in the invasion and metastasis of prostate cancer cells.

Previous reports have noted that the inhibition of PI3K activity hinders all platelet-derived growth factor (PDGF)-induced lamellipodial motile processes in fibroblasts, including extension, peripheral ruffling, and dorsal ruffle formation [19]. Because PI3K is involved in the early stage of signal transduction from the PDGF receptor in fibroblasts [21], all responses to PDGF could be intercepted by PI3K inhibition. However, PI3K activity is reportedly unnecessary for M-CSF-induced ruffling and EGF-induced dorsal ruffling in A431 cells [22], [23]. Thus, signaling from distinct receptors leads to ruffle formation in various cell types. In our experiments using the optogenetic control of Rac1 activity, we directly induced Rac1-mediated lamellipodial activity without upstream signaling from receptors. Because the involvement of PI3K in early signal transduction from different types of receptors could therefore be ignored, we could elucidate the role of PI3K in lamellipodial motility downstream of Rac1. Using live-cell imaging combined with PA-Rac1 photomanipulation, we could clearly demonstrate that the lamellipodium first extends outward parallel to the substratum, and that the fully extended lamellipodium then shows ruffling activity by curling up the leading edge. Furthermore, we found that the lamellipodial extension induced by PA-Rac1 activation was severely perturbed by PI3K inhibitors while peripheral ruffling was not inhibited. These results suggest that two types of lamellipodial motility, extension and ruffling, are differentially regulated by PI3K-dependent and PI3K-independent signaling pathways.

Wiskott-Aldrich syndrome protein (WASP) and WASP-family verprolin-homologous protein (WAVE) family proteins are activators of Arp2/3-dependent polymerization [17]. WAVE family proteins are associated with lamellipodial formation through the Rac1 signaling pathway. To prevent disordered actin polymerization, WAVE family proteins exist as heteropentameric protein complexes that hinder their own active sites. Although WAVEs are functionally activated by GTP-bound Rac1 when actin polymerization is initiated, WAVEs cannot bind directly to GTP-bound Rac1. Instead, IRSp53 (insulin receptor tyrosine kinase substrate p53) works as a linker molecule to connect Rac1 and the WAVE complex [18]. GTP-bound Rac1 induces an allosteric change in the WAVE complex that exposes its active site; WAVE2 then activates the Arp2/3 complex, which becomes a nucleus for actin polymerization at the leading edge of the lamellipodium [24]–[26].

In our PA-Rac1-activation experiments, WAVE2 was localized to the leading edges of extending lamellipodia. However, neither WAVE2 recruitment nor lamellipodial extension was observed when PI3K activity was inhibited. These results suggest that PI(3,4,5)P_3_ is required for WAVE2 recruitment and for lamellipodial extension. Because WAVE2 has a PI(3,4,5)P_3_-binding sequence [19], PI(3,4,5) P_3_ may recruit WAVE2 to the leading edge. Suetsugu et al. [18] reported that the activity of the WAVE2 complex was optimized by IRSp53 in association with activated Rac1 and PI(3,4,5)P_3_. Furthermore, the simultaneous binding of GTP-bound Rac and acidic phospholipids such as PI(3,4,5)P_3_ to WAVE2 is required for the efficient recruitment and activation of WAVE2 [17]. These previous reports strengthen our assertion that the inhibition of lamellipodial extension by PI3K inhibitors results from the perturbation of WAVE2 recruitment.

In this study, the activation of PA-Rac1 induced the production of PI(3,4,5)P_3_ and the recruitment of WAVE2 when lamellipodial extension was initiated. Furthermore, PI3K inhibitors hindered the recruitment of WAVE2 and PI(3,4,5)P_3_ and suppressed lamellipodial extension. These findings indicate that PI3K plays an essential role in initiating lamellipodial extension. Furthermore, we employed constitutively active Rac1Q61L-expressing cells to observe the response of the extended lamellipodia to the inhibition of PI3K activity. In cells expressing Rac1Q61L, the extended lamellipodia were relatively resistant to PI3K inhibitors. In addition, the PI3K inhibitors actually enhanced the peripheral ruffling activity of the lamellipodia, but rather enhanced this activity. Thus, while PI3K may not be crucial for the maintenance of extended lamellipodia or for ruffling activity, the initiation of lamellipodial extension is highly dependent on PI3K. Notably, PI3K inhibitors also enhanced peripheral ruffling activity in Rac1Q61L-expressing PC-3 cells, although the mechanism for this phenomenon remains unclear. Using EGF-stimulated A431 cells, we have previously shown that PI(4,5)P_2_ is enriched in the membrane of ruffles, however, PI(3,4,5)P_3_ levels are elevated only at the closing of circular ruffles into macropinosomes [23]. Therefore, ruffle formation is likely more dependent on PI(4,5)P_2_. Because PI3K inhibition results in an increase in PI(4,5)P_2_ levels, it may be hypothesized that this increased PI(4,5)P_2_ enhances ruffling activity. We recently reported that the sequential breakdown of PI(3,4,5)P_3_ is also important for the completion of macropinosome formation from membrane ruffling [27]. Thus, the roles of phosphoinositide metabolism in membrane ruffling and lamellipodial extension are more complicated and important than we previously predicted. Future studies should conduct more detailed examinations of the interactions of each phosphoinositide with its effectors and/or other signaling pathways.

Recently, the overexpression of a Rac1 activator protein (14-3-3 protein zeta) and several GEFs (VAV3, P-Rex1) was identified in prostate cancer [28]–[30]. Moreover, castration-resistant prostate cancer cells, which have a high malignant potential associated with invasion and metastasis, overexpress Rac1 [31]. These findings suggest that Rac1 overexpression affects the progression of prostate cancer. Although several studies have shown that PI3K inhibitors obstruct the migration of prostate cancer cells as induced by chemical mediators, those studies assumed that PI3K affected the upstream signal transduction of Rac1 [32]–[34]. To our knowledge, no previous report has examined the relationship between PI3K and Rac1 downstream signal transduction in prostate cancer. In this study, we clearly showed that PI(3,4,5)P_3_ and the Rac1 downstream effector protein WAVE2 act in a coordinated manner in lamellipodial extension, which contributes to the migration of prostate cancer cells. Therefore, the inhibition of PI3K activity effectively obstructs the Rac1-overexpression-mediated migration of prostate cancer cells.

## Conclusions

Optogenetic technology enabled us to spatiotemporally control PA-Rac1 activity in prostate cancer cells. We demonstrated that the inhibition of PI3K activity suppressed lamellipodial extension but had less of an inhibitory effect on peripheral ruffling. The present study indicates that PI3K, acting downstream of Rac1, has an essential role in the initiation of lamellipodial extension, which underlies prostate cancer cell invasion and metastasis. The better understanding and further characterization of the molecular regulation of the lamellipodial motile processes of metastatic prostate cancer cells will provide new insights for the development of cancer therapies.

## Supporting Information

Figure S1 **Effects of other PI3K inhibitors on lamellipodial extension induced by photoactivation.** PC-3 cells were transiently transfected with pTriEx/mCherry-PA-Rac1. The cells were subjected to repeated photoactivation in the absence (control) or presence of 100 nM wortmannin or 1 µM ZSTK474. The leading edge of the extending lamellipodium is outlined in red. Both wortmannin and ZSTK474 obstructed lamellipodial extension. Scale bars, 10 µm.
(TIF)

Figure S2 **PA-Rac1-induced lamellipodial extension was not influenced by dimethyl sulfoxide.** PC-3 cells were transiently transfected with pTriEx/mCherry-PA-Rac1 and subjected to local photoactivation of PA-Rac1 (rectangular area outlined by blue dots). The cells were subjected to repeated photoactivation in the absence (control) or presence of 0.1% dimethyl sulfoxide (DMSO). Kymographic analysis was performed at a line placed across a lamellipodium. After 30 min of treatment with 0.1% DMSO, the cell showed lamellipodial extension to the same extent as in the absence of DMSO. Scale bars, 10 µm.
(TIF)

Movie S1 **Photoactivation of PA-Rac1 induces lamellipodial extension and subsequent ruffling.** This movie shows that local PA-Rac1 activation induced lamellipodial extension and subsequent ruffling. PC-3 cells were transiently transfected with pmCherry-PA-Rac1 (shown in red). The 445-nm laser-irradiated area is indicated with a blue rectangle. This movie corresponds to the images shown in Fig. 1. Scale bar, 10 µm.
(MP4)

Movie S2 **PI3K is required for lamellipodial extension but not for peripheral ruffling.** This movie shows that the lamellipodial extension induced by PA-Rac1 activation was suppressed by LY294002. The PA-Rac1 signal is shown as red. The 445 nm laser-irradiated area is indicated with a blue rectangle. This movie corresponds to the images shown in Fig. 3A. Scale bar, 10 µm.
(MP4)

Movie S3 **Effect of LY294002 on the extended lamellipodial motility in PC-3 cells expressing constitutively active Rac1Q61L.** This movie shows that the extended lamellipodium is not shortened but is actively ruffled by PI3K inhibition. PC-3 cells were transiently transfected with pmCitrine-Rac1Q61L (shown in green). This movie corresponds to the images shown in Fig. 6. Scale bar, 10 µm.
(MP4)
