# Supplementary figures and images for: Rac1-Dependent Lamellipodial Motility in Prostate Cancer PC-3 Cells Revealed by Optogenetic Control of Rac1 Activity
Source: PLoS One. 2014 May 21;9(5):e97749. doi: 10.1371/journal.pone.0097749 (PMC4029798; doi:10.1371/journal.pone.0097749)

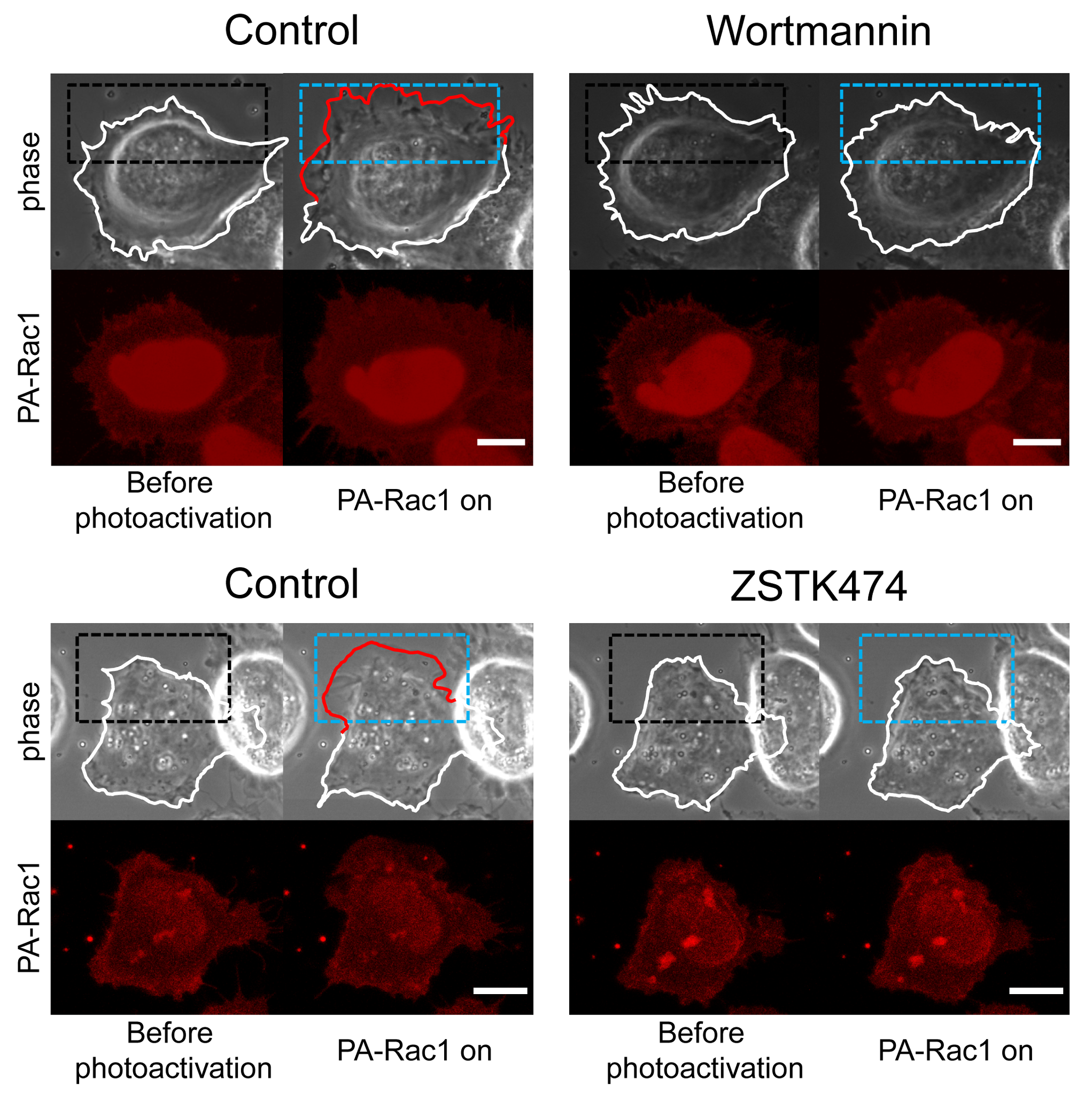

Supplement: Figure S1 — Effects of other PI3K inhibitors on lamellipodial extension induced by photoactivation. PC-3 cells were transiently transfected with pTriEx/mCherry-PA-Rac1. The cells were subjected to repeated photoactivation in the absence (control) or presence of 100 nM wortmannin or 1 µM ZSTK474. The leading edge of the extending lamellipodium is outlined in red. Both wortmannin and ZSTK474 obstructed lamellipodial extension. Scale bars, 10 µm. (TIF) [file pone.0097749.s001.tif]

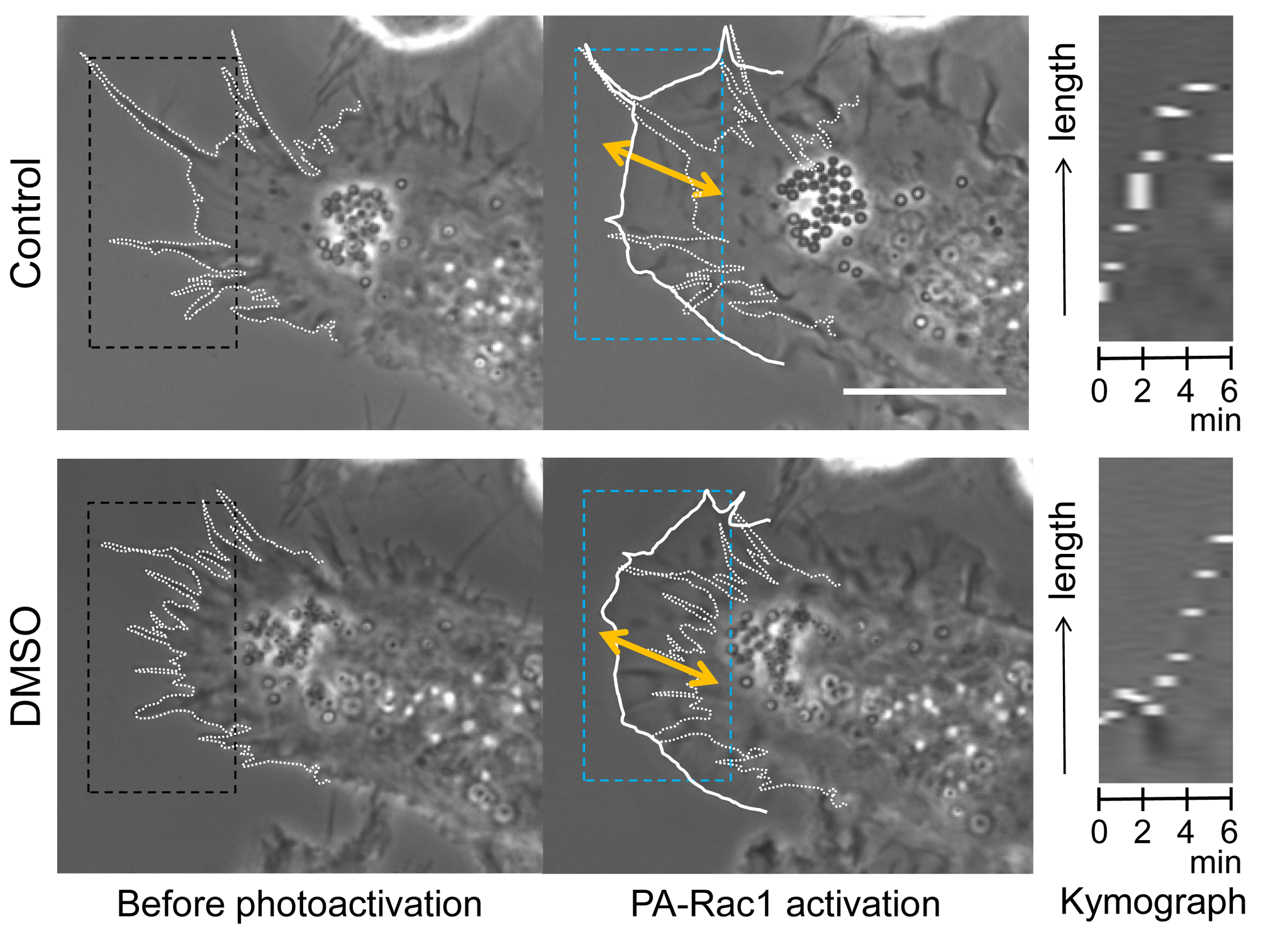

Supplement: Figure S2 — PA-Rac1-induced lamellipodial extension was not influenced by dimethyl sulfoxide. PC-3 cells were transiently transfected with pTriEx/mCherry-PA-Rac1 and subjected to local photoactivation of PA-Rac1 (rectangular area outlined by blue dots). The cells were subjected to repeated photoactivation in the absence (control) or presence of 0.1% dimethyl sulfoxide (DMSO). Kymographic analysis was performed at a line placed across a lamellipodium. After 30 min of treatment with 0.1% DMSO, the cell showed lamellipodial extension to the same extent as in the absence of DMSO. Scale bars, 10 µm. (TIF) [file pone.0097749.s002.tif]
